# Peer review of "Drug Discovery Strategies for Inherited Retinal Degenerations"

_biology, 2022, doi:10.3390/biology11091338_

Round 1

Reviewer 1 Report

The review written by Das and Imanishi focuses on Drug discovery strategies for inherited retinal diseases. The manuscript covers a good landscape of the current approach. However, some parts are quite unbalanced between Photoreceptor part and Retinal ganglion cells (e.g. absence of iPSC-based approach in the photoreceptor section). Some aspects to improve the current version are indicated below.

General comment:

As a general improvement, adding extra information (maybe as a table) about the animal models mentioned in the review indicating, at least, the disease, the onset and the current therapeutics conducted in such models, could help the readers to understand the relevance of them in either photoreceptor or RGC strategies.

Specific comments per section:

Abstract:

The authors indicate “those disorders primarily cause death of photoreceptor cells or retinal ganglion cells (RGCs)”. However, in some IRD, RPE cells are also lost (even earlier than photoreceptors). As a consequence, the nourishment requirement of the photoreceptors cannot be maintained resulting in subsequent photoreceptor lost.

Introduction:

The whole manuscript is well-cited or examples are well provided. However, in line 33, the authors mentioned “various visual disorders are caused by loss of photoreceptor cells or RGCs”. The authors must discern between one cause and the other and at least mention one specific disease for each cause and refer it properly.

The current aim of the review needs to be clarified: “This review focuses on the development of small molecule therapies” and add “for pathway modulation”. Otherwise, it can be misunderstood with other small molecules like ASO that are mutation specific. Despite in the following sentences indicate that “small molecule therapies are often aimed at modulating general pathways afflicted in multiple genetic disorders and have potential to treat a broader spectrum of blinding diseases”, there is no a clear statement which indicates that these molecules are the aim of this review.

Targets and drug discoveries for photoreceptor degeneration:

1.    As many aspects of this section also include or specifically refer to RPE, the authors should include this also in the title either indicating “photoreceptor and RPE degeneration” or adding “and RPE dysfunction”.

2.    Figure 1. Sections A and B are complicated to follow up. Figures are complicate to relate with the information given by the legend. Adding a more descriptive representation is needed.

3.    In line 194, the authors summarized the main outcomes of the antagonist and inhibitor drugs in the Abca4-/- mouse model. The authors have focused on the successful blocking on the accumulation of A2E and lipofuscin, and then mentioned that these inhibitors are on clinical development in other diseases. However, reference 74 only refer the use of an RPE65 inhibitor in the Abca4-/- mouse model, and it does not include any other functional study either in AMD or diabetic retinopathies. It is true that in the discussion of reference paper 74 these diseases are mentioned, but this reference does not support the sentence “those compounds have been in various stages of clinical development for Stargardt disease, age-related macular degeneration, and diabetic retinopathy”. Please, include more references about the use of these drugs in these disorders or replace the reference for one more accurate.

4.    The authors dedicated one paragraph to discuss the use of SIRT6 inhibitors and suggest the use of these drugs in “retina degeneration models”. Could the authors elaborate more this suggestion, highlighting which models they refer to or if it’s any model already being used to study SIRT6 also applied to study the SIRT6 inhibitors?

5.    Stem cell-based models for drug development are included in the section about RGC, but it is totally missed in this section.

Targets and drug discoveries for RGC degeneration:

1.    Clarify the term “they’’ in line 433. Who does it term refer to? To the authors on paper 148 or somebody else? If it refers to that authors I highly recommend to mention the author’s name before.

2.    Figure 2.

a.    Add the biological sources from the cells obtained from patients (e.g. blood, fibroblast, etc).

b.    Indicate how long the RGC differentiation takes

c.    Only IP injection is conducted with the iPSC-derived RGC?

Concluding remarks:

I found the concluding remark concise and easy to follow. However, I miss a further discussion not only in cell-based models, but also about animal model based approaches mentioned in the paper. In addition, USHA2A and EYS are only mentioned in this section but nothing about current drug development or treatment strategies for these genes, despite its size, is being conducted. Therefore, it is needed to complete or provide any information about them in the main text. Otherwise, the authors need to focus the remarks on points already addressed in the main text.

Author Response

Comments and Suggestions for Authors

The review written by Das and Imanishi focuses on Drug discovery strategies for inherited retinal diseases. The manuscript covers a good landscape of the current approach. However, some parts are quite unbalanced between Photoreceptor part and Retinal ganglion cells (e.g. absence of iPSC-based approach in the photoreceptor section). Some aspects to improve the current version are indicated below.

Thank you very much for insightful and detailed comments that were helpful to improve this review manuscript. The goal of this review is to cover topics relevant to small molecule development. Regarding iPSC approaches, therefore, we did not cover some of the excellent studies about transplantation of iPSC-derived RPE, photoreceptor-like cells or retinal ganglion cells. Likewise, there has been excellent studies done on antisense oligonucleotides and gene therapies for IRDs. To clarify the focus of this manuscript, we added the following sentence to the manuscript.

Lines 110 -111: “For antisense oligonucleotide, gene, and stem cell therapies, we recommend excellent reviews published recently [38-43].”

Regarding the use of iPSC for drug screening (absence of iPSC-based approach in the photoreceptor section), it is difficult to culture differentiated photoreceptor cells in isolation, and thus, we did not extensively cover this aspect in the photoreceptor section of this review. Application of retinal organoids for drug screening is in its infancy. Fortunately, retinal ganglion cells, derived from stem cells, can be purified, and cultured well for reliable drug screening. The first author of this manuscript is an expert in this area. Thus, we extensively covered this topic in the section “Targets and drug discoveries for RGC degeneration”. Although premature, iPSC technologies will eventually become more amenable for drug screening in context to photoreceptor or RPE degenerative conditions as seen in inherited blindness. Therefore, we modified the following part to elaborate possible future application of such technology.

Lines 665 - 667: “Stem cells can be genetically engineered so that these differentiated photoreceptors express green fluorescent protein, which can be utilized to assess photoreceptor survival in response to small molecule treatments [211].”

General comment:

As a general improvement, adding extra information (maybe as a table) about the animal models mentioned in the review indicating, at least, the disease, the onset and the current therapeutics conducted in such models, could help the readers to understand the relevance of them in either photoreceptor or RGC strategies.

In response to the comment, we provided additional detailed information about the animal models mentioned throughout the manuscript. Additional citations were provided to clarify the sources of the information. Below indicate the revised portions of the main text:

Lines 124 – 131: “PDE6 consists of α-, β-, γ- subunits and mutations of PDE6 β-subunit gene are found in retina degeneration 1 (rd1) and 10 (rd10) mouse models of retinitis pigmentosa [46]. For preclinical studies, use of rd1 is limited by its extremely early onset of photoreceptor degeneration, which occurs prior to eye opening and becomes extensive prior to the time of drug administrations. Partly because rd10 mouse demonstrates relatively slow degeneration of photoreceptors [46], it is one of the most commonly used inherited retinal degeneration (IRD) models to test investigational drugs.”

Lines 211 – 213: “Abca4 knockout mouse serves as a model for testing therapeutics, because it demonstrates accumulation of A2E and lipofuscin in RPE cells [70], potentially leading to very slow degeneration of photoreceptors in this model [59].”

Lines 229 – 235: “An unbiased systems pharmacology approach was introduced to identify novel G protein-coupled receptor (GPCR) targets, adrenoceptor α 2C (Adra2c) and serotonin receptor 2a (Htr2a), for treating Abca4-/- Rdh8-/- double knockout mouse [80] which is a Stargardt disease model demonstrating light-induced rapid photoreceptor degeneration [81]. This light-dependent nature allowed efficient testing of GPCR ligands in promoting photoreceptor survival.”

Lines 279 – 280: “Metformin promotes photoreceptor survival in rd10 mice [94, 95].”

Lines 283 – 288:  “SIRT6 is a potential target for IRD because glycolysis in photoreceptors is promoted by genetically suppressing this protein, as demonstrated in a recessive IRD mouse model with Pde6bH620Q/H620Q mutation [96, 97]. Several SIRT6 inhibitors have been developed and tested in cancer and diabetes animal models [98], and thus it might be interesting to test them in retina degeneration models, starting from mice with mutations in Pde6 genes such as rd10 and Pde6bH620Q/H620Q.”

Lines 325 – 327: “PBA also acts as a chemical chaperone and improves vision of R91W RPE65 knock-in mouse [104], which is characterized by compromised 11-cis-retinal production and early retinal dysfunction [118].”

Lines 328 – 331: “PBA also demonstrated efficacy in the transgenic mouse model of primary open angle glaucoma caused by a gain of function missense mutation in the myocilin gene (Tg-MYOCY437H), leading to misfolding of the protein product in the trabecular meshwork cells and IOP elevation [99].”

Lines 343 – 347: “More recently, non-retinoid chaperones of rhodopsin mutants have been investigated and demonstrated success in mitigating photoreceptor loss in P23H rhodopsin gene heterozygote knock-in mouse (RhoP23H/+), which is a model of adRP and shows progressive photoreceptor degeneration starting around P20 [127, 128].”

Lines  367 – 369: “Those efforts resulted in a novel small molecule BF844, which successfully mitigated sensory loss in an USHIII animal model which demonstrated progressive hearing loss from P22 - 70 [129].”

Lines 382 – 384: “…and prevention of photoreceptor degeneration in RhoP23H/+ mice and transgenic rats expressing P23H mutant rhodopsin gene [133].”

Line 448 – 451: “At 6 months, OptnE50K homozygous knock-in mice without treatment showed nearly 15% decrease in RGC layer thickness while amlexanox treated mice showed only 3% decrease compared to the wild-type mice [153]”

Line 676 – 679: “For glaucomatous RGC degeneration, several new genetically modified animal models have been developed. These models are useful for preclinical studies of small molecules because the onset and duration for their blinding conditions were extensively characterized (for details, please read [213])”  

Specific comments per section:

Abstract:

The authors indicate “those disorders primarily cause death of photoreceptor cells or retinal ganglion cells (RGCs)”. However, in some IRD, RPE cells are also lost (even earlier than photoreceptors). As a consequence, the nourishment requirement of the photoreceptors cannot be maintained resulting in subsequent photoreceptor lost.

Thanks for the comment. It is true that RPE atrophy is observed prior to the onsets of photoreceptor degeneration in some IRDs, such as Stargardt disease. Following changes are made to the abstract:

Lines 18 – 20: “Those disorders frequently cause death of photoreceptor cells or retinal ganglion cells. In a subset of these disorders, photoreceptor cell death is a secondary consequence of retinal pigment epithelial cell dysfunction or degeneration.”

Introduction:

The whole manuscript is well-cited or examples are well provided. However, in line 33, the authors mentioned “various visual disorders are caused by loss of photoreceptor cells or RGCs”. The authors must discern between one cause and the other and at least mention one specific disease for each cause and refer it properly.

We agree that the statement in line 33 was confusing, because it was not clear what is causing what. We made the following edits to address the concern:

Lines 44 – 46: “Various visual disorders are characterized by loss of photoreceptor cells or RGCs. For example, photoreceptor cells and RGCs gradually die in retinitis pigmentosa and optic neuropathies, respectively.”

The current aim of the review needs to be clarified: “This review focuses on the development of small molecule therapies” and add “for pathway modulation”. Otherwise, it can be misunderstood with other small molecules like ASO that are mutation specific. Despite in the following sentences indicate that “small molecule therapies are often aimed at modulating general pathways afflicted in multiple genetic disorders and have potential to treat a broader spectrum of blinding diseases”, there is no a clear statement which indicates that these molecules are the aim of this review.

ASOs (antisense oligonucleotides) are relatively large (typically > 6000 Daltons) and not considered small molecules (less than 900 - 1000 Daltons). Thus, ASOs are not discussed in this review. To clarify the focus of this review, following part was edited with an additional citation for ASO therapy targeting IRDs:

Lines 108 - 111: “Ocular tissue is amenable to various interventions including small molecules, antisense oligonucleotides, adeno-associated viruses (AAVs), and stem cells. For antisense oligonucleotide, gene, and stem cell therapies, we recommend excellent reviews published recently [38-43].”

Targets and drug discoveries for photoreceptor degeneration:

  1. As many aspects of this section also include or specifically refer to RPE, the authors should include this also in the title either indicating “photoreceptor and RPE degeneration” or adding “and RPE dysfunction”.

As pointed out here, various therapeutic molecules target the pathways in RPE cells. Those targeting RPE cells do not necessarily prevent RPE degeneration or address RPE dysfunction. For example, visual cycle modulators make RPE cells less functional (by decreasing the amount of retinoid engaged in the visual cycle) to prevent the toxic buildup of bisretinoid. To accommodate the aspects of RPE biology covered in this review, we revised the title of the section as follows:

Line 117 – 118: “Targets and drug discoveries for modulating pathways in photoreceptor and retinal pigment epithelial cells.”

  1. Figure 1. Sections A and B are complicated to follow up. Figures are complicate to relate with the information given by the legend. Adding a more descriptive representation is needed.

Thanks for the comments. We agree that the original figure legend was difficult to follow. We added more descriptions to indicate where in the figure correspond to specific descriptions in the text. Followings are the revised texts:

Lines 162 – 177: “(A) Drug screenings can be conducted using stable mammalian cell lines that express unstable mutant proteins of interest fused to antibody epitope tags or fluorescent proteins. Using fluorescence intensity (green) as a surrogate, it is possible to measure quantities of mutant proteins. Such assay can be combined with high-throughput fluorescence microscopy to assess the efficacies of drug-like molecules in stabilizing specific proteins. Protein stabilization will result in increased fluorescence (shown in green). (B) An assay was designed to detect and screen out proteasome inhibitors. Red fluorescent protein fused to a strong degron motif was genetically introduced to a mammalian cell line (red). As the degron motif promotes proteasome-mediated degradation, this red fluorescent protein exists at low quantities (left, red). The same cell line was designed to express unstable mutant protein tagged with a fluorescent protein (green) which also exists at low quantities as explained in (A). The cells will demonstrate increased red and green fluorescence when proteasomes are inhibited (right top, red and green stripes). When a drug can stabilize mutant proteins specifically without inhibiting proteasome, the cells will only demonstrate increased green fluorescence (right bottom, green). In principle, similar assays can be developed for any mutated proteins of interest.”

  1. In line 194, the authors summarized the main outcomes of the antagonist and inhibitor drugs in the Abca4-/- mouse model. The authors have focused on the successful blocking on the accumulation of A2E and lipofuscin, and then mentioned that these inhibitors are on clinical development in other diseases. However, reference 74 only refer the use of an RPE65 inhibitor in the Abca4-/- mouse model, and it does not include any other functional study either in AMD or diabetic retinopathies. It is true that in the discussion of reference paper 74 these diseases are mentioned, but this reference does not support the sentence “those compounds have been in various stages of clinical development for Stargardt disease, age-related macular degeneration, and diabetic retinopathy”. Please, include more references about the use of these drugs in these disorders or replace the reference for one more accurate.

Thanks for pointing out incomplete citation. To refer to the clinical development of these molecules, we’ve added ClinicalTrials.gov Identifiers for each indication (Stargardt disease, age-related macular degeneration, diabetic retinopathy). We also added a few citations where applicable. Following edits were made to the main text:

Lines 220 – 223: “Those compounds [72] have been in various stages of clinical development for Stargardt disease (NCT04489511 and NCT05244304) [77, 78], age-related macular degeneration (NCT03845582), and diabetic retinopathy (NCT02753400) [36, 78].”

  1. The authors dedicated one paragraph to discuss the use of SIRT6 inhibitors and suggest the use of these drugs in “retina degeneration models”. Could the authors elaborate more this suggestion, highlighting which models they refer to or if it’s any model already being used to study SIRT6 also applied to study the SIRT6 inhibitors?

Thanks for the comments. To clarify the animal models used in this line of studies, following changes were made to the main text:

Lines 283 – 288: “SIRT6 is a potential target for IRD because glycolysis in photoreceptors is promoted by genetically suppressing this protein, as demonstrated in a recessive IRD mouse model with Pde6bH620Q/H620Q mutation [96, 97]. Several SIRT6 inhibitors have been developed and tested in cancer and diabetes animal models [98], and thus it might be interesting to test them in retina degeneration models, starting from mice with mutations in Pde6 genes such as rd10 and Pde6bH620Q/H620Q.”

  1. Stem cell-based models for drug development are included in the section about RGC, but it is totally missed in this section.

Please see our response at the beginning of this document.

Targets and drug discoveries for RGC degeneration:

  1. Clarify the term “they’’ in line 433. Who does it term refer to? To the authors on paper 148 or somebody else? If it refers to that authors I highly recommend to mention the author’s name before.

We agree that the pronoun “They” could refer to various preceding nouns. In the original text, “They” referred to the small molecules with known interactions with the mitochondrial proteins (MPs) and beneficial effect on mitochondria as discussed in [158]. We have clarified as stated below.

Line 475 – 477: “These small molecules could be investigated to identify RGC protective compounds for developing glaucoma therapy.”

  1. Figure 2.
  2. Add the biological sources from the cells obtained from patients (e.g. blood, fibroblast, etc).
  3. Indicate how long the RGC differentiation takes
  4. Only IP injection is conducted with the iPSC-derived RGC?

Thanks for the suggestions. We have incorporated them in the figure 2 legend. In the scheme, iPSC-derived RGCs are not injected via IP. Hit molecules, identified through drug screening efforts, are administered through IP injection. We have made changes in the figure and corresponding legend. Following is the revised legend.

Line 581 – 491: “RGCs differentiated from iPSCs which are derived from skin fibroblasts or peripheral blood mononuclear cells of patients. During days 35-45 of differentiation, RGCs (red cells) could be isolated by immunopurification. These RGCs provide a platform for drug screening against disease associated mutations. HTS based on microscopic analysis could identify drugs that improve cellular morphology, mitochondrial morphology and lower ROS level. Plate-reader based assays could identify drugs that increase cell viability and lower apoptosis. These compounds are referred to as hits. As a next step, RGC protective hits need to be tested in animal models such as by intraperitoneal (IP) injection in mice for identifying safe dose and treatment duration regimens, then efficacy for protecting RGCs under injury such as optic nerve crush (ONC). This strategy could pave the path for clinical trials in human with prior data for optimum dose and toxicity in human cells”.

Concluding remarks:

I found the concluding remark concise and easy to follow. However, I miss a further discussion not only in cell-based models, but also about animal model based approaches mentioned in the paper. In addition, USHA2A and EYS are only mentioned in this section but nothing about current drug development or treatment strategies for these genes, despite its size, is being conducted. Therefore, it is needed to complete or provide any information about them in the main text. Otherwise, the authors need to focus the remarks on points already addressed in the main text.

Thanks for the comment. In the revised manuscript, we extended the discussion of animal models, focusing on recent development in creating large animal models of IRDs, and genetic models of glaucoma. We have added the following discussion:

Line 671 – 679: “In addition to advance in generating retinal organoids, there are encouraging developments in generating new animal models of inherited retinal degenerations. For example, a pig model of Usher syndrome type IC, which demonstrates visual impairments and morphological defects in photoreceptor cells, was developed [212]. Because of the physiological similarities to human, pig models are more favorable than rodents for preclinical testing of investigational therapies. For glaucomatous RGC degeneration, several new genetically modified animal models have been developed. These models are useful for preclinical studies of small molecules because the onset and duration for their blinding conditions were extensively characterized (for details, please read [213]).”

We agree that discussions of USH2A and EYS were tenuous. The discussions of USH2A and EYS were removed from the concluding remarks. The issue regarding the size limit for AAV packaging has been extensively discussed in previously published reviews.

We hope that the above responses have addressed the reviewer#1’s comments and the manuscript is acceptable for publication in Biology.

Submission Date

04 August 2022

Date of this review

16 Aug 2022 19:10:10

Reviewer 2 Report

This interesting review aims to summarize ongoing efforts on strategies for neglected degeneration diseases and promote new ideas on therapeutics developments.  I have the following comments/advice for authors to consider. 

(1) For the assay proposed in figure 1.  To propose new assays targeting PKG inhibitor discovery, cellular phenotypic assay usually is not the first choice due to low specificity to the desired drug target, in-vitro enzymatic kinase assay usually provides a higher target specificity screen. It is advisable to propose an enzymatic screen followed by a cellular phenotypic assay for small molecular discovery. 

(2) Line 201, for treating blinding disorders, may be worth adding a few sentences discussing cell-based GPCR assay on Adra2c and serotonin receptor 2a. 

(3) Line 316. Typically overexpression of a protein prone to be degraded is difficult to be engineered into a stable cell line. It is advisable to add additional considerations into assay design, such as the inducible promoter for target protein expression and optimizing the target protein expression level for the compound screen. 

(4) Line 541. It may be useable to add discussion of patient-derived ipscs.

(5) Line 599. It may be useable to add discussion of proposed assays on stem cell-derived organoids for drug discovery.  

Author Response

Comments and Suggestions for Authors

This interesting review aims to summarize ongoing efforts on strategies for neglected degeneration diseases and promote new ideas on therapeutics developments.  I have the following comments/advice for authors to consider. 

We are pleased that you found this review to be interesting. Below please find our point-by-point responses to your comments.

(1) For the assay proposed in figure 1.  To propose new assays targeting PKG inhibitor discovery, cellular phenotypic assay usually is not the first choice due to low specificity to the desired drug target, in-vitro enzymatic kinase assay usually provides a higher target specificity screen. It is advisable to propose an enzymatic screen followed by a cellular phenotypic assay for small molecular discovery. 

Thanks for the comment. In this review, we are not proposing new assays for discovering new PKG inhibitors. The goal of this review is to introduce recent studies discovering PKG inhibitors by François Paquet-Durand’s group. They initially designed cGMP analogs with expectations these molecules will modulate cGMP-dependent pathways. Then they conducted phenotypic assays to directly test the efficacies of these molecules in promoting survival of degenerating photoreceptors. Following studies confirmed that a few of these efficacious molecules indeed have effects consistent with PKG inhibition. This study is mentioned from lines 151 – 160 of the main text. Enzymatic assays are often ideal as the primary screening methods; however, those assays cannot detect molecules with inadequate pharmacokinetics parameters (e.g. optimal cell penetration, etc.). Indeed, various clinical developments fail because of suboptimal pharmacokinetics properties of investigational drugs. Thus sometimes, starting from phenotypic assays provide significant benefits over in vitro enzymatic assays as reviewed in this manuscript.

(2) Line 201, for treating blinding disorders, may be worth adding a few sentences discussing cell-based GPCR assay on Adra2c and serotonin receptor 2a. 

Thanks for a great point. To direct the readers to recent exciting developments in GPCR pharmacology, following edits were made to the main text:

Lines 244 – 249: “As mentioned above for photoreceptor degeneration caused by phototransduction defects, these studies on a Stargardt model recapitulate the pivotal roles of cyclic nucleotides and GPCRs in various photoreceptor degenerative conditions [81]. Structure-based and cell-based approaches have been combined to discover molecules that target and modulate GPCRs [85, 86]. We envision such combined approaches can further improve the selectivity of GPCR ligands for treating IRDs.”

(3) Line 316. Typically overexpression of a protein prone to be degraded is difficult to be engineered into a stable cell line. It is advisable to add additional considerations into assay design, such as the inducible promoter for target protein expression and optimizing the target protein expression level for the compound screen. 

Thanks for the comment. It is difficult to overexpress proteins that are prone to be degraded in a stable cell line, however, it is very easy to engineer a transgenic stable cell line expressing mRNA encoding such proteins. The purpose of this engineering is not to overexpress the protein, but to express mRNA encoding unstable protein which is expected to be degraded; however, if a small molecule were to be able to stabilize these translated proteins, the cell line will show increased protein expression as shown in Figure 1. We’ve edited the following sentence to clarify the point:

Lines 352 – 354: “In the first step of this strategy, mammalian transgenic cells stably expressing mRNA encoding CLRN1N48K tagged with an antibody epitope was developed and treated with various small molecules.”

(4) Line 541. It may be useable to add discussion of patient-derived ipscs.

We incorporated the strength of patient-derived iPSCs in developing RGC protective drugs. These changes are shown in below main text.

Line 598 – 602: “Differentiated hRGCs from patient derived induced pluripotent stem cells (iPSCs) provide unique advantage for identifying disease mechanisms and developing drugs relevant not only to the disease-causing mutations but also to patient-specific genetic backgrounds (Figure 2). This approach is key for developing precision medicine personalized for a patient.”

(5) Line 599. It may be useable to add discussion of proposed assays on stem cell-derived organoids for drug discovery.  

The following sentence was added to discuss an assay utilizing retina organoids for drug discovery:

Lines 665 – 667: “Stem cells can be genetically engineered so that these differentiated photoreceptors express green fluorescent protein, which can be utilized to assess photoreceptor survival in response to small molecule treatments [211].”

We hope that the revised manuscript and above responses have addressed the reviewer#2’s comments and it is acceptable for publication in Biology.

Submission Date

04 August 2022

Date of this review

18 Aug 2022 16:38:23
